# Effect of Axial Clearance Variation on Dual-Rotor Flowmeter Performance

**DOI:** 10.3390/s24134389

**Published:** 2024-07-06

**Authors:** Fuji Huang, Liang Yan, Chabi Christian Monsia, Yuxiang Han, Jiabao Liu, Hao Zan

**Affiliations:** 1 School of Energy and Power Engineering, Jiangsu University of Science and Technology, Zhenjiang 212100, China; 13039668800@163.com (F.H.); monsiachristian@gmail.com (C.C.M.);; 2 Beijing LiDaZhuo Fire Control Technology Co., Ltd., Beijing 100049, China

**Keywords:** turbine flowmeter, entropy generation, axial clearance, pressure loss

## Abstract

This study examines the impact of axial clearance variations on the performance characteristics of a dual-rotor flowmeter (DRT-FM) through numerical simulations, with the validity of the numerical results verified by calibration experiments. The results indicate that within the range of 200 L/h to 1600 L/h, the K factors of different groups increase as clearance increases. The K factor of the 0.80 mm group is the largest, showing an average increase of around 6% compared to that of the 0.50 mm group. Additionally, Linearity E also decreased, with a minimum of 1.07% in the 0.65 mm group, significantly lower than the 3.33% in the 0.50 mm group. Furthermore, the pressure loss increased slightly, with the 0.65 mm group having the largest pressure loss; however, at a flow rate of 1600 L/h, the pressure loss only increases by 0.186 kPa compared to that of the 0.50 mm group. Flow field analysis reveals that changes in axial clearance predominantly affect pressure distribution. Larger clearances reduce low-pressure regions on upstream and downstream transition surfaces, thereby reducing energy loss due to pressure changes. Entropy analysis further demonstrates that higher axial clearance decreases energy loss in the upstream and downstream stationary domains, optimizing the DRT-FM’s energy characteristics.

## 1. Introduction

With the rapid development of the aviation industry, the demand for high-precision fuel flow measurement in harsh conditions has significantly increased. Fuel flow, as a core technical parameter of aviation engines, is directly related to engine speed, thrust, and other important parameters, and is often used as a control quantity in the control system of aviation engines for real-time control of engines [1,2]. The limited internal space of aeroengines imposes strict requirements on flowmeters regarding volume, accuracy, and reliability. A turbine flowmeter, because of its simple and reliable structure and high measurement accuracy, can measure liquids and gases and other characteristics, and is favored by the aviation industry [3,4].

A turbine flowmeter is a volumetric flowmeter. When the fluid flows through the turbine flowmeter, its rotor is rotated by force, and its rotational speed is proportional to the flow rate, and a pulse signal proportional to the flow rate is generated through the sensor [5]. Early research on turbine flowmeters focused on theoretical models of turbine flowmeter response characteristics [6,7,8,9]. However, these models are unable to obtain relevant information about the internal flow field of the turbine flowmeter, and the phenomena such as flow separation, cyclone, and reflux are still unexplained. With the development of technology, new tools are available for the study of the internal flow field of a turbine flowmeter. A high-speed camera was used to visualize the internal flow field of a turbine flowmeter at low flow rates [10], and the results give a response curve of the turbine flowmeter at low flow rates, which is divided into three phases: non-responsive, unevenly rotating, and steadily rotating. With the development of computational fluid dynamics, the accuracy and reliability of numerical calculation have been improved, and numerical calculation methods have been widely used in the study of the internal flow field of turbine flowmeters [11,12,13,14]. A turbine flowmeter is essentially a rotating machine, and clearance variations are a common factor affecting the performance of rotating machines. It has been found that the variations in the turbine flowmeter blade tip clearance can affect the flowmeter’s rotational speed, resulting in a deviation in flowmeter measurement [15], and influences the responsiveness of the flowmeter and the start-up flow rate at low flow rates [16]. There is an axial clearance between the turbine flowmeter’s guide vane and rotor, where flow leakage is easy to produce; at the same time, the tail flow of the guide vane will also have an impact on the rotor, and the turbine flowmeter axial clearance variations will also have an impact on the flow measurement [17,18].

The dual-rotor flowmeter is a novel turbine flowmeter. Compared with the conventional turbine flowmeter, the dual-rotor flowmeter, known for its high precision, wide range, vibration resistance, and other advantages for an aerospace flowmeter, is an ideal choice, and has a broad application prospect. The dual-rotor flowmeter model utilizing two coupled rotors for flow measurement was first proposed by Lee et al. [19]. The accuracy of the flow calculation method of the dual-rotor flowmeter was verified by calibration experiments [20]. Through calibration experiments on a 12.5 mm diameter dual-rotor flowmeter in the 200:1 flow span range and 42:1 dynamic viscosity span range, [21], the experimental results of the calibration work show that the dual-rotor flowmeter is not sensitive to the flow medium, and its high precision and wide range deepen the understanding of the measurement characteristics of the dual-rotor flowmeter; the proposed Extended Lee model was verified, providing a theoretical basis for the optimization of the dual-rotor flowmeter. The dynamic response process, the flow characteristics of the flow field at low flow rates, and the pressure pulsation characteristics of the dual-rotor flowmeter have been investigated [22,23]. The results have found that the upstream rotor is affected by the wake of the leading flow; there is a localized high-vorticity distribution at the upstream rotor outlet and the downstream rotor inlet; the pressure fluctuation of the dual-rotor flowmeter mainly comes from the interaction of the rotors, with the maximum amplitude at the downstream rotor inlet; and the variation in the blade tip clearance affects the rotational speed of the rotors of the dual-turbine flowmeter.

The variation in axial clearances impact dual-rotor flowmeter performance, yet research on this effect is rare. The study first uses a calibration experiment to verify the effectiveness of the numerical calculation. By adjusting axial clearance, we analyze its effects on the flow and energy characteristics of the dual-rotor flowmeter, providing a reference for optimal dual-rotor flowmeter design.

## 2. Numeric Simulation Scheme

### 2.1. Geometry Description

**Figure 1:**
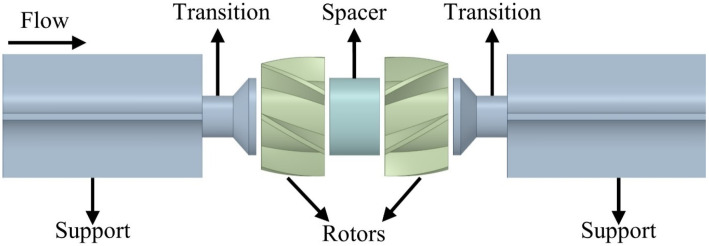
Internal structure of a DRT-FM.

A dual-rotor flowmeter is a volumetric flowmeter. Like conventional turbine flowmeters, the measured fluid passes through the rotor blades, driving the rotors. The sum of the rotation speeds of the two rotors is proportional to the volumetric flow, generating a pulse signal proportional to the rotation speed. The internal structure of a dual-rotor flowmeter features a pair of rotors connected in parallel, with symmetrical transitions and supports, as shown in Figure 1.

### 2.2. Goveneration Equations

The flowing operating fluid has no significant temperature increase and can be approximated as an incompressible fluid. There is no heat exchange during the flow process, and the energy equation can be ignored. Therefore, the basic control equation in the Cartesian coordinate system is as follows:

(1) $$\frac{\partial u_{i}}{\partial x_{i}}=0$$

(2) $$\frac{\partial u_{i}}{\partial t}+u_{j}\frac{\partial u_{i}}{\partial x_{j}}=-\frac{1}{\rho }\frac{\partial p}{\partial x_{i}}+\nu \frac{\partial^{2}u_{i}}{\partial x_{j}\partial x_{j}}$$

where *u_i_* and *u_j_* are the velocity component in coordinate system; *x_i_* and *x_j_* are the coordinate component; *p* is the pressure; *ρ* is the fluid density; and *ν* is the kinematic viscosity.

The commercial software FLUENT is used. This study involves high pressure gradients, high rotor speeds, and high flow velocities. Considering the influence of turbulent shear stress and the demand of accurate calculation of flow separation, shear stress transport (SST) *k*–ω was selected in this research. The transmission equation can be expressed by Equation (3).

(3) $$\mu_{t}=\frac{\rho \alpha_{1}k}{\max\left(\alpha_{1}\omega ,SF_{2}\right)}$$

The turbulent kinetic energy can be expressed by Equation (4).

(4) $$\;\;\frac{\partial }{\partial t}(\rho k)+\frac{\partial }{\partial x_{j}}(\rho U_{j}k)=\frac{\partial }{\partial x_{j}}\left[(\mu +\mu_{t}\sigma_{k})\frac{\partial k}{\partial x_{j}}\right]+P_{k}-\beta^{*}k\omega$$

The turbulent frequency is as follows.

(5) $$\begin{array}{l} \frac{\partial }{\partial t}(\rho \omega )+\frac{\partial }{\partial x_{j}}[\rho U_{j}\omega -(\mu +\mu_{t}\sigma_{\omega })\frac{\partial \omega }{\partial x_{j}}]\\ =\alpha \rho S^{2}-\beta \rho \omega^{2}+2(1-F_{1})\rho \sigma_{\omega 2}\frac{1}{\omega }\frac{\partial k}{\partial x_{j}}\frac{\partial \omega }{\partial x_{j}} \end{array}$$

*P_k_*, Blending Function *F*_1_ and *F*_2_, and their relevant equations are shown in Equations (6)–(11).

(6) $$F_{1}=\tanh(\arg_{1}^{4})$$

(7) $$F_{2}=\tanh(\arg_{2}^{2})$$

(8) $$\arg_{1}=\min\left(\max\left(\frac{\sqrt{k}}{\beta^{*}\omega y},\frac{500\nu }{y^{2}\omega }\right),\frac{4\sigma_{\omega 2}\rho k}{CD_{k\omega }y^{2}}\right)$$

(9) $$\arg_{2}=\max\left(\frac{2\sqrt{k}}{\beta^{*}\omega y},\frac{500\nu }{y^{2}\omega }\right)$$

(10) $$CD_{k\omega }=\max\left(2\rho \sigma_{\omega 2}\frac{1}{\omega }\nabla k\nabla \omega ,10^{-10}\right)$$

(11) $$P_{k}=\mu_{t}\frac{\partial U_{i}}{\partial x_{j}}(\frac{\partial U_{i}}{\partial x_{j}}+\frac{\partial U_{j}}{\partial x_{i}})$$

where *μ* and *μ_t_*, respectively, represent the dynamic viscosity and turbulent dynamic viscosity. *S* is the absolute value of the vorticity. *y* represents the mesh height of the first layer near the wall. The constants *α*, *β*, *σ_k_*, and *σ_ω_* are computed by a blend from the corresponding constants via *α* = *α*_1_*F*_1_ + *α*_2_(1 − *F*_1_), etc. The constants for this model are as follows: β* = 0.09, *α*_1_ = 5/9, *β*_1_ = 0.075, *σ_k_*_1_ = 0.85, *σ_ω_*_1_ = 0.5, *α*_2_ = 0.44, *β*_2_ = 0.0828, *σ_k_*_2_ = 1, and *σ_ω_*_2_ = 0.856 [24].

### 2.3. Gird Generation and Grid Independence Test

**Figure 2:**
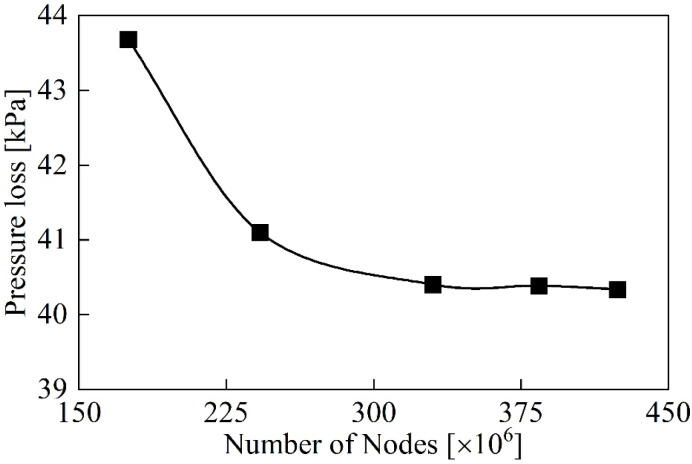
Grid independency study for different numbers of nodes.

The CATIA software is used for modeling, and tetrahedral unstructured mesh is used for grid generation. The number of grids is important for the results of numerical calculations. Five sets of different numbers of grids were generated for the 0.50 mm groups: 1.73 million, 2.42 million, 3.30 million, 3.84 million, and 4.24 million. To select a suitable grid, pressure loss of the DRT-FM is compared. The grid independence verification results are shown in Figure 2.

**Figure 3:**
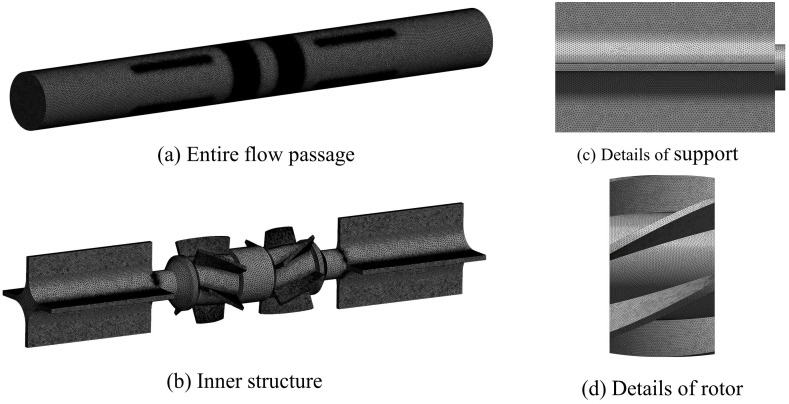
Grids details of the DRT-FM.

As the number of grids increases, the pressure loss decreases. When the number of grids is more than 3.30 million, the fluctuation value of the total pressure loss is less than 1%. Considering the calculation sources, required time, and demand for fine mesh, a set of 3.84 million grids was selected. Grid encryption is performed in areas of high curvature on the rotor blades, bumps on the support edges, and structural discontinuities to accurately capture detailed flow field characterizes. The average quality of the mesh cells is above 0.8, which satisfies the calculation requirements of the turbulence model. The internal structure of the grid details is shown in Figure 3.

### 2.4. Flowmeter Performance Indicators

Every time the rotating rotor blades passes through the sensor of the turbine flowmeter, an electronic pulse is generated. The meter factor *K* of the turbine flowmeter is defined as the ratio of the frequency of rotor to the volume flow rate through the flowmeter, which is one of the important indicators of the performance of the turbine flowmeter. For the DRT-FM, the *K* factor can be expressed by Equation (12).

(12) $$K_{i}=\frac{\left(f_{up}+f_{down}\right)}{Q_{v}}\times 3600$$

where *K_i_* is the *K* factor for different flow rate points, 1/L; *f_up_* and *f_down_* are the frequencies of the upstream and downstream rotors, Hz; and *Q_v_* is the flow rate, L/h.

Because of the mechanical resistance of the bearing and fluid resistance and other effects, the *K* factor cannot be maintained as a constant; making the *K* factor close to the constant is important to improve the performance of the flowmeter. Linearity *E* is one of the important indicators of turbine flowmeter performance. The linearity *E* can be expressed by Equation (13).

(13) $$E=\frac{{\left(K_{i}\right)}_{\max}-{\left(K_{i}\right)}_{\min}}{{\left(K_{i}\right)}_{\max}+{\left(K_{i}\right)}_{\min}}\times 100\%$$

where (*K_i_*)_max_ is the maximum value of the *K* factors; (*K_i_*)_min_ is the maximum value of the *K* factors. Within the flow range, a smaller *E* indicates greater stability and better performance of flowmeter.

### 2.5. Entropy Theory

According to the second law of thermodynamics, entropy production is a parameter that characterizes mechanical energy loss due to irreversible factors in the process of energy conversion. In simulations with the Reynolds time-averaged equation method, entropy production is induced by two parts: one is by the time-averaged velocity gradient and the other by the turbulent loss induced by the velocity fluctuation. The local entropy production rate caused by velocity gradient can be expressed by Equation (13).

(14) $$\overset{•}{S_{D}^{\prime \prime \prime }}=\overset{•}{S_{\bar{D}}^{\prime \prime \prime }}+\overset{•}{S_{D^{\prime }}^{\prime \prime \prime }}$$

(15) $$\begin{array}{ll} \overset{•}{S_{\bar{D}}^{\prime \prime \prime }}= & \frac{\mu_{m}}{T}\left[{\left(\frac{\partial {\bar{u}}_{2}}{\partial x_{1}}+\frac{\partial {\bar{u}}_{1}}{\partial x_{2}}\right)}^{2}+{\left(\frac{\partial {\bar{u}}_{3}}{\partial x_{1}}+\frac{\partial {\bar{u}}_{1}}{\partial x_{3}}\right)}^{2}+{\left(\frac{\partial {\bar{u}}_{2}}{\partial x_{3}}+\frac{\partial {\bar{u}}_{3}}{\partial x_{2}}\right)}^{2}\right]-\\ & \frac{2}{3}\frac{\mu_{m}}{T}{\left(\frac{\partial {\bar{u}}_{1}}{\partial x_{1}}+\frac{\partial {\bar{u}}_{2}}{\partial x_{2}}+\frac{\partial {\bar{u}}_{3}}{\partial x_{3}}\right)}^{2}+2\frac{\mu_{m}}{T}\left[{\left(\frac{\partial {\bar{u}}_{1}}{\partial x_{1}}\right)}^{2}+{\left(\frac{\partial {\bar{u}}_{2}}{\partial x_{2}}\right)}^{2}+{\left(\frac{\partial {\bar{u}}_{3}}{\partial x_{3}}\right)}^{2}\right] \end{array}$$

(16) $$\begin{array}{ll} \overset{•}{S_{D^{\prime }}^{\prime \prime \prime }}= & \frac{\mu_{m}}{T}\left[{\left(\frac{\partial u_{2}^{\prime }}{\partial x_{1}}+\frac{\partial u_{1}^{\prime }}{\partial x_{2}}\right)}^{2}+{\left(\frac{\partial u_{3}^{\prime }}{\partial x_{1}}+\frac{\partial u_{1}^{\prime }}{\partial x_{3}}\right)}^{2}+{\left(\frac{\partial u_{2}^{\prime }}{\partial x_{3}}+\frac{\partial u_{3}^{\prime }}{\partial x_{2}}\right)}^{2}\right]-\\ & \frac{2}{3}\frac{\mu_{m}}{T}{\left(\frac{\partial u_{1}^{\prime }}{\partial x_{1}}+\frac{\partial u_{2}^{\prime }}{\partial x_{2}}+\frac{\partial u_{3}^{\prime }}{\partial x_{3}}\right)}^{2}+2\frac{\mu_{m}}{T}\left[{\left(\frac{\partial u_{1}^{\prime }}{\partial x_{1}}\right)}^{2}+{\left(\frac{\partial u_{2}^{\prime }}{\partial x_{2}}\right)}^{2}+{\left(\frac{\partial u_{3}^{\prime }}{\partial x_{3}}\right)}^{2}\right] \end{array}$$

where *T* is the local temperature; *μ_m_* is the turbulent eddy viscosity; *u_i_* (*i* = 1.2.3) represents the time averaged velocity; *u*^′^*_i_* (*i* = 1.2.3) represents the fluctuating velocity.

As fluctuating velocities are not directly measurable, researchers have related the entropy production rate induced by turbulent velocity fluctuations to the *ε* or *ω* terms in turbulent models. For the *k*–*ω* model, the local entropy production rate induced by velocity fluctuation is given by Equation (16) [25].

(17) $$\overset{•}{S_{D^{\prime }}^{\prime \prime \prime }}=\beta \frac{\rho_{m}k\omega }{T}$$

where *β* = 0.09; *ω* is the turbulent frequency, *s*^−1^; and *k* is the turbulent kinetic energy, *m*^2^/*s*^2^.

Research shows that that there is a large deviation in calculating the direct dissipative entropy production rate near the wall. Therefore, local entropy in the region from the wall to the first grid is calculated.

(18) $$\overset{•}{S_{w}^{\prime \prime \prime }}=\frac{\vec{\tau }\cdot \vec{v}}{T}$$

where $\vec{\tau }$ is the wall shear stress in the wall, Pa; $\vec{v}$ is the velocity of the first grid near the wall, m/s. The total entropy production in the flow filed can be obtained by the volume or the surface integration of the local entropy production rate.

(19) $$\overset{•}{S}=\overset{•}{S_{\bar{D}}}+\overset{•}{S_{D^{\prime }}}+\overset{•}{S_{w}}=\int_{V}\overset{•}{S_{\bar{D}}^{\prime \prime \prime }}dv+\int_{V}\overset{•}{S_{D^{\prime }}^{\prime \prime \prime }}dv+\int_{A}\overset{•}{S_{w}^{\prime \prime \prime }}da$$

Finally, the total energy loss in the system can be obtained by Equation (20).

(20) $$I=I_{\bar{D}}+I_{D^{\prime }}+I_{W}=\int_{V}\overset{•}{S_{\bar{D}}^{\prime \prime \prime }}dvdt+\int_{V}\overset{•}{S_{D^{\prime }}^{\prime \prime \prime }}dvdt+\int_{A}\overset{•}{S_{w}^{\prime \prime \prime }}dad$$

### 2.6. Numerical Calculation Strategy

**Figure 4:**
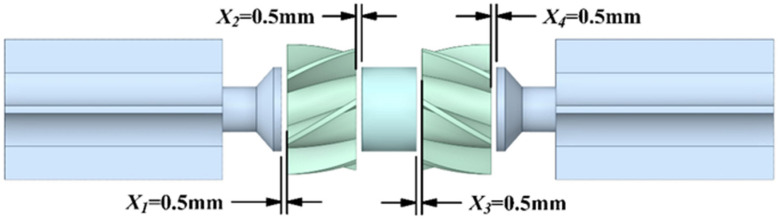
The structure of 0.50 mm group.

The DRT-FM has four axial clearances between the rotors and the transition and spacer, all of which are of the same size. This paper studies the effect of axial clearance changes on the performance of the DRT-FM, and provides a reference for the optimization of the DRT-FM. In this paper, a DRT-FM is designed for three groups with different axial clearances. The dimensions of the axial clearance are 0.50 mm, 0.65 mm, and 0.80 mm. The schematic diagram of the 0.5 mm group is shown in Figure 4, and the values of the four clearances in each group are the same.

**Figure 5:**
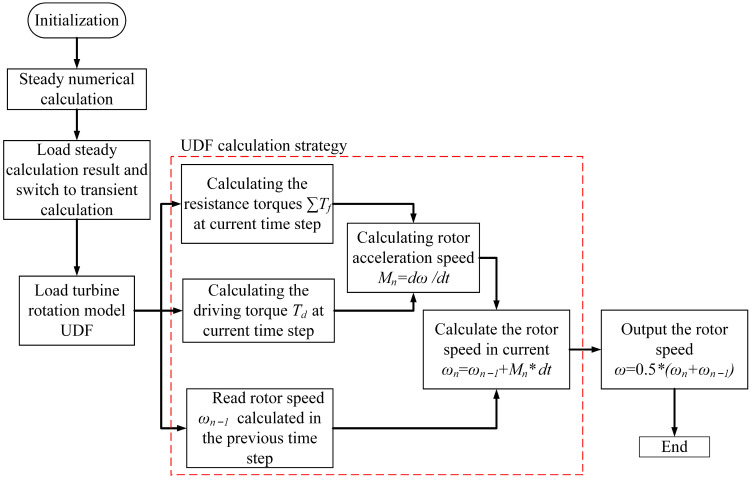
Numerical calculation strategy.

The boundary conditions for numerical calculations are velocity inlet and pressure outlet, respectively, and the coupled solution is performed using the SIMPLEC method. Firstly, the flow field of the DRT-FM is calculated in steady state; the result of the steady state calculation is used as the initial value of the flow field in the transient calculation. The equilibrium rotational speed of the rotor is obtained by an automatic iterative method at different flow rates by means of an external program that realizes the free impact of the fluid on the surface of the rotor blades when performing transient calculations. The numerical calculations strategy is shown in Figure 5.

## 3. Experimental Results and Simulation Verification

**Figure 6:**
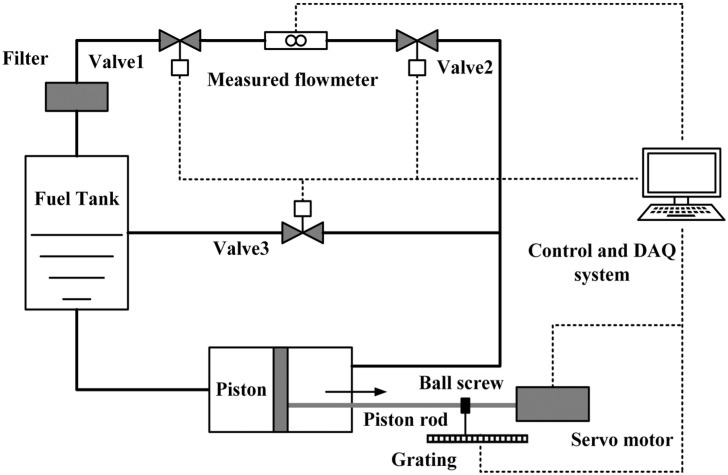
Schematic illustration of active volumetric flow calibration device.

The calibration of flowmeters is mainly carried out using methods such as the mass method, volumetric method, and volumetric tube method, with priority given to using volumetric tube flow calibration devices. The calibration experiment platform is shown in Figure 6. Before performing calibration, valve 1 and valve 2 are opened, and valve 3 is closed; the servo motor drives the piston to move in the active volumetric tube through a ball screw to generate a standard flow source. The liquid passes through valve 1, then flows through the measured flowmeters and ultimately into the fuel tank. The volumetric flow rate is obtained by multiplying the piston stroke measured by the grating with the piston planal area and dividing by the time required for the flow. The calibration of the flowmeters is achieved by comparing the reading value of the measured flowmeters with the standard volumetric flow value measured by the volumetric tube.

**Figure 7:**
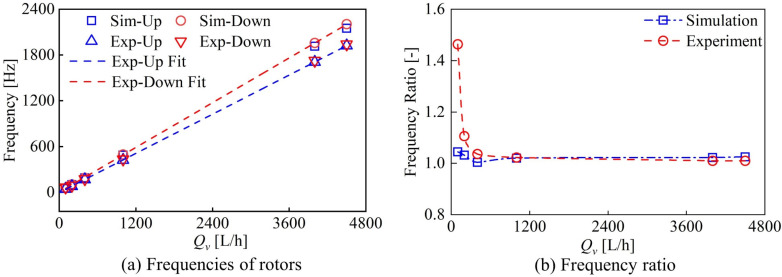
Comparison of the rotor frequency and frequency ratio between experiments and simulations.

Calibration experiments were conducted by randomly measuring seven points within the flow range of 200 L/h to 4500 L/h, and the average value of each flow point measured was used as the final data for comparison with numerical calculation results, as shown in Figure 7. The variation trend of rotor frequency with flow rate is consistent, and the frequencies of upstream and downstream rotors are higher than the experimental values. The frequency ratio of the rotor in the numerical calculation (downstream rotor frequency/upstream rotor frequency) is much lower than that of the rotor in the experiment below 1200 L/h. With the increase in flow rate, the frequency ratio of the rotor in the numerical calculation matches well with the frequency ratio of the rotor in the experiment above 1200 L/h. The frequency ratio of the numerical calculation is slightly higher than the experimental value.

The numerical calculation neglects the actual friction and magnetic resistance of the bearing to the rotor motion, which results in the rotor frequency of numerical calculation being higher than the experimental results and makes the frequency ratio of numerical calculation deviate from the experimental results at the low flow rate. However, with the increase in flow rate, these effects are gradually weakened, and the numerical results are more consistent with the experimental results.

## 4. Result and Discussion

Transient numerical calculations of the DRT-FM under various clearances are conducted. The effect of changes in axial clearances on the DRT-FM is studied by analyzing its performance characteristics, internal flow field, and energy loss.

### 4.1. Comparative Analysis of Performance Characteristics

**Figure 8:**
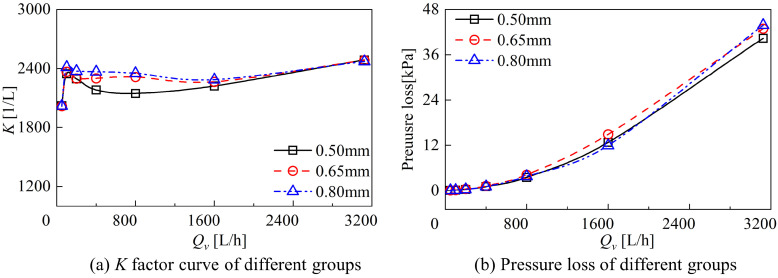
Comparison of the flowmeter performance of different groups.

The variation in the K factor and pressure loss with flow rate for different groups are compared in Figure 8. The K factor and linearity E in the range of 200 L/h to 1600 L/h are presented in Table 1, whereas the pressure loss of different groups is displayed in Table 2. Due to the fluid drag effect, the K factor of different groups appears as a hump zone at low flow rates. After the hump zone, the variation in the K-factor of each group is more stable and shows good measurement performance in the range of 200 L/h to 1600 L/h. The K factor of the different groups increases at high flow rates. With the increase in axial clearance, in the flow range of 200 L/h to 1600 L/h, the K-factor of 0.80 mm group is the largest, and its average K factor is increased by 6.02% compared with 0.50 mm group. With the increase in axial clearance, the linearity E of different groups decreased, where the linearity E of the 0.65 mm group was the minimum at 1.07%, the linearity of the 0.80 mm group was 1.98%, and the linearity of the 0.50 mm group was the maximum at 3.33%. The increase in axial clearance increases the K factor of the DRT-FM, while decreasing the linearity, improving its performance.

Pressure loss is one of the indicators to evaluate the performance of the flowmeter, and reducing the pressure loss can reduce the energy dissipation during the use of the flowmeter. As the flow rate increases, the pressure loss of the DRT-FM under different clearances obviously rises, and the pressure loss with the flow rate of different groups shows similar trends. At a range of 200 L/h–1600 L/h, with the increase in clearance, the pressure loss of the DRT-FM increases and then decreases, and the pressure loss of the 0.65 mm group is the largest. The pressure loss of the DRT-FM increases with the increase in axial clearance in the high flow rate of 3100 L/h, and the pressure loss of the 0.80 mm group is the largest so far, which is 43.798 kPa.

Although the increase in axial clearance increases the pressure loss of the DRT-FM, the increase in axial clearance increases the rotational speed of the rotors of the DRT-FM, increases the K-factor, and decreases the linearity E, which results in an improvement in the measurement performance of the dual-rotor flowmeter.

### 4.2. Comparative Analysis of Internal Flow Characteristics

**Figure 9:**
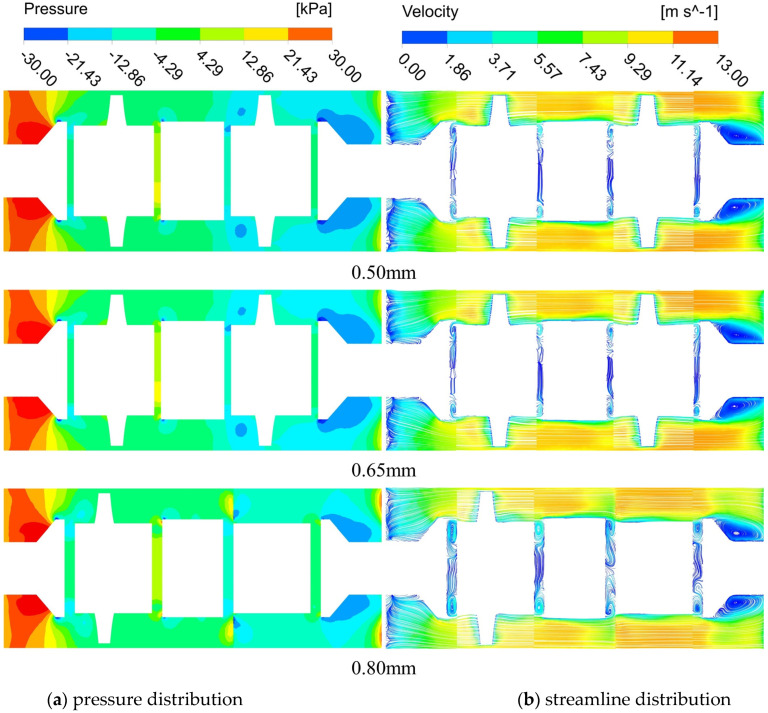
Comparison of the flow flied at y = 0 mm plane.

To analyze the effect of axial clearance variation on the DRT-FM, the internal flow field of different groups of DRT-FMs is analyzed. The flow distribution at the y = 0 mm plane and the pressure distribution for the 0.50 mm, 0.65 mm, and 0.80 mm groups are compared in Figure 9. The velocity field distribution at this cross-section appears similar for each group, as shown in Figure 9a.

A localized reflux phenomenon occurs when part of the fluid impinges on the tapering region of the upstream transition, creating a high-velocity zone near the upstream rotor. Here, the fluid velocity comprises both the mainstream and rotor-induced velocities. This combined effect, along with throttling, generates a high-velocity zone that extends downstream. The extent of this zone diminishes in the middle and rear of the spacer domain but reappears due to the influence of the downstream rotor. The gradual expansion of the flow channel in the downstream transition leads to flow separation, creating a backflow zone and resulting in energy dissipation. Changes in the axial clearance minimally impact the overall velocity field of the DRT-FM, affecting only a portion of the fluid within the clearance. Increasing the axial clearance enhances the vortex structure, influencing the pressure loss of the DRT-FM but having a negligible effect on the overall pressure loss.

The pressure distribution of the DRT-FM with varying clearances is illustrated in Figure 9b. A low-pressure region is observed on the surface of the upstream transition, extending downstream and affecting the upstream rotor. Low-pressure regions are also present on both sides of the spacer; their distribution is influenced by the rotors and extends downstream. In the downstream transition, a broad distribution of low-pressure regions is noted, accompanied by vortex formation and backflow, which significantly contribute to the pressure loss in the DRT-FM. As the axial clearance increases, the low-pressure region in the downstream transition diminishes, leading to an increase in pressure within the flow channel, thus reducing the pressure loss in this area.

**Figure 10:**
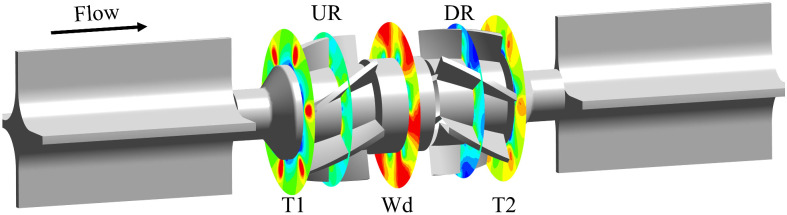
The section plane distribution in flow fields.

To investigate the effect of axial clearance variation on the pressure inside the DRT-FM, five XY sections were created inside the DRT-FM, and the locations of the sections are shown in Figure 10. Here, T1 is the plane at the end of the upstream transition, the UR plane is the plane in the middle of the upstream rotor, Wd is the plane in the middle of the spacer, DR is the plane in the middle of the downstream rotor, and T2 is the plane at the front end of the downstream transition.

**Figure 11:**
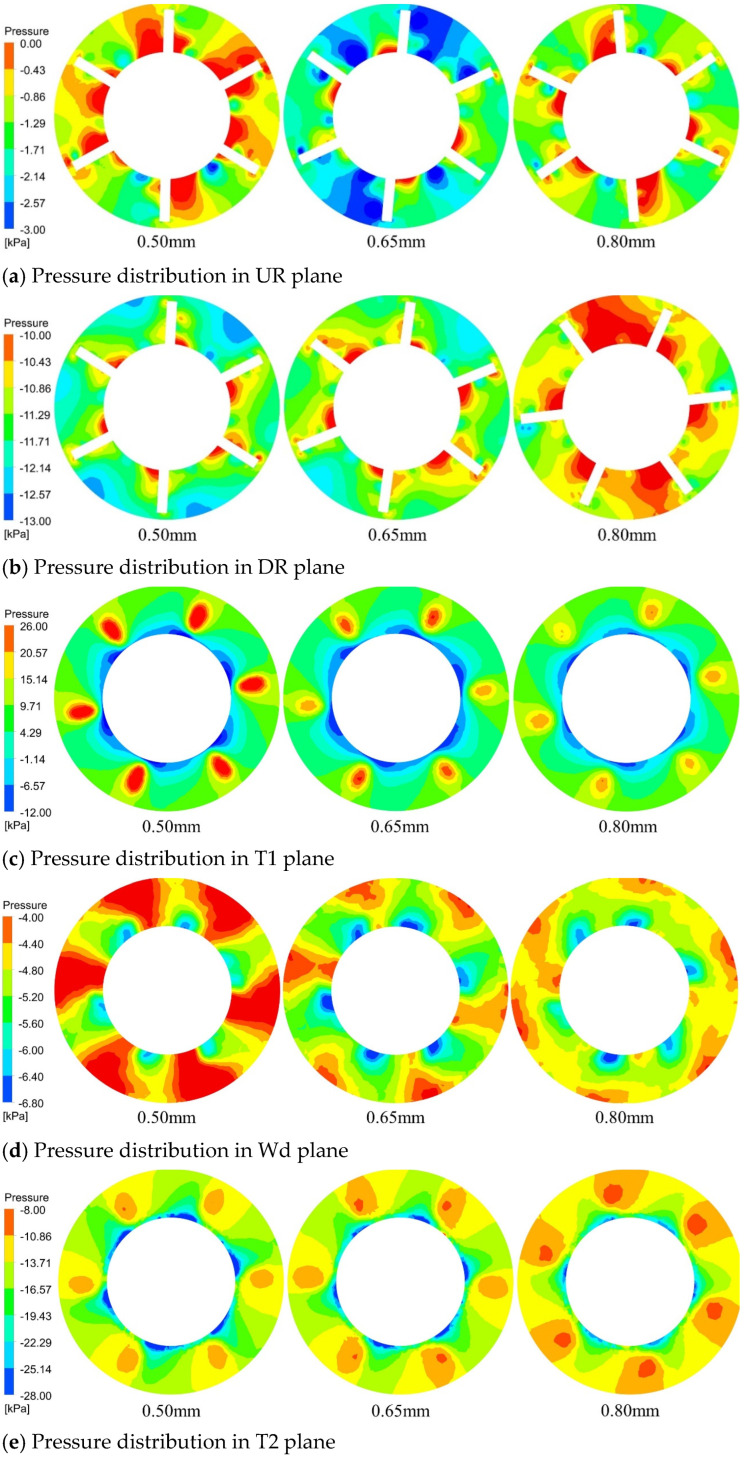
Pressure distribution in rotor domain.

The pressure distributions at the section planes for different groups are compared in Figure 11. There is little difference in the pressure distribution at the UR plane across the groups. At the UR plane, a low-pressure region is present on the suction surface side of the blades, and a dramatic pressure change occurs on the hub surface, leading to significant energy dissipation. In the 0.65 mm group, increasing the axial clearance significantly decreases the pressure at the suction surface of the blades, expanding the low-pressure region. This greatly affects rotor speed and energy loss. Further increasing the axial clearance, the pressure on the suction surface in the 0.80 mm group recovers but remains lower than the pressure at this location in the 0.50 mm group.

At the DR plane, the pressure is lower than at the UR plane. The pressure in the flow channel of the DR plane is significantly reduced, and the range of the low-pressure region increases markedly. Increasing the axial clearance significantly reduces the low-pressure distribution in the downstream rotor domain, mitigates the dramatic pressure changes on the hub surface, and improves the performance of the DRT-FM.

At the T1 plane, located at the end of the upstream transition, a low-pressure region exists on its surface, while a high-pressure zone is distributed in the middle of the flow path due to the upstream rotor rotation. As the axial clearance increases, the low-pressure region on the surface decreases, the influence of upstream rotor rotation diminishes, and both the magnitude and extent of the high pressure in the flow channel decrease.

The Wd plane, positioned in the middle of the spacer, experiences a complex flow influenced by both the upstream and downstream rotors. A low-pressure region is present near the wall surface, while a high-pressure region, affected by rotor rotation, exists within the flow channel. Increasing the axial clearance reduces the influence of rotor rotation, decreases near-wall pressure, expands the low-pressure region, and decreases the high pressure in the flow channel.

At the T2 plane, situated in front of the downstream transition, the pressure distribution across different groups is similar. A low-pressure region is found on the surface of the downstream transition, and a high-pressure region is present in the middle of the flow channel due to downstream rotor rotation. Increasing the axial clearance reduces the extent of the low-pressure region on the surface, raises the pressure in the middle of the flow channel, and stabilizes the pressure distribution within the flow channel. Thus, increasing the axial clearance can reduce the low-pressure distribution on both upstream and downstream transition surfaces, thereby reducing pressure loss.

### 4.3. Comparative Analysis of Energy Loss Based on Entropy Production Theory

According to the second law of thermodynamics, entropy production is a parameter that characterizes the loss of mechanical energy due to irreversible factors during energy conversion. Mechanical energy is not only converted into pressure and kinetic energy, but also inevitably some of it will be converted into internal energy in the flow field. This kind of mechanical energy being converted into internal energy is unavoidable. Entropy production analysis allows for a quantitative evaluation of the energy losses in a DRT-FM with different clearances and the distribution of the energy losses in space. The entropy production in the equations in Section 2.5 is divided into the EPDD (entropy production rate by direct dissipation), EPTD (entropy production rate by turbulent dissipation), and EPWS (entropy production rate by wall shear).

**Figure 12:**
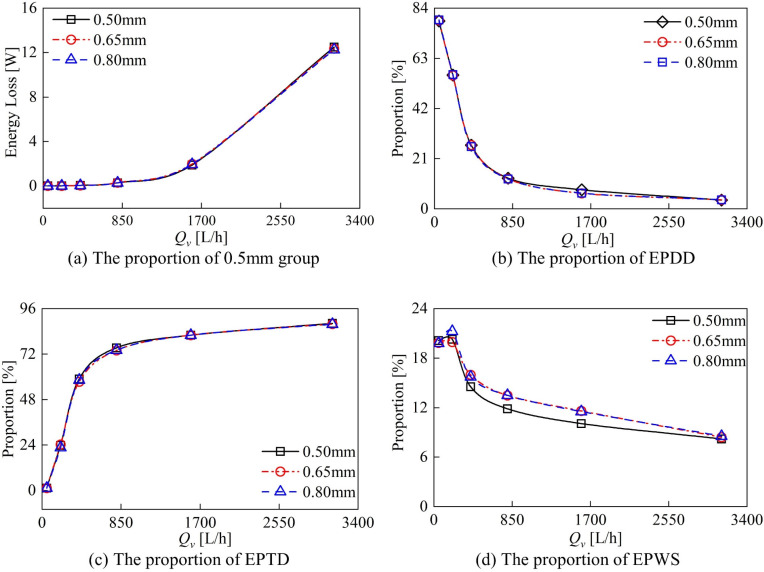
Comparison of the proportion of different energy losses.

The variations in different types of entropy generation and energy loss with flow rate for different groups are compared in Figure 12. The variation in energy loss with flow rate for different groups is compared in Figure 12a. The variation in energy loss with flow rate for different groups is compared in Figure 12a and Table 3. The variation in the clearances did not have a significant effect on the energy loss of the DRT-FM. In the flow range of 200 L/h to 1600 L/h, the energy loss of the DRT-FM increases and then decreases with the increase in the axial clearance, and the energy loss of the 0.65 mm group is the largest. In the high flow rate of 3100 L/h, the increase in axial clearance will make the energy loss of the DRT-FM decrease, and the energy loss of the 0.80 mm group is the lowest. The trend of energy loss with flow rate is consistent with the trend of pressure loss with flow rate of the DRT-FM, and the distribution pattern is similar, as shown in Figure 7. The energy loss of the DRT-FM can be quantitatively analyzed by entropy production analysis.

At low flow rates, EPDD has the largest proportion, followed by EPWS, with EPTD being the smallest. With the increase in flow rate, both the proportion of EPDD and EPWS decreases, while the proportion of EPTD rises gradually. At high flow rates, the proportion of EPTD is the largest, followed by EPWS, and the proportion of EPDD is the smallest. The results indicate that entropy production resulting from the velocity gradient predominantly contributes to the energy loss in the flow field within the DRT-FM. At low flow rates, the internal flow field of the DRT-FM is in a laminar flow state, so the energy loss caused by EPTD accounted for a small percentage, around 2%; EPDD caused by the energy loss accounted for a large percentage, around 80%. With the increase in flow, the DRT-FM internal flow field Reynolds number rises, and gradually it transitions to a turbulent state; the EPTD caused by the proportion of energy loss increases, to around 85%, and the EPDD caused by the proportion of energy loss decreases, to around 5%. The proportion of energy loss caused by EPWS is larger at low flow rates, around 20%; its proportion decreases as the flow rate increases, and the proportion of energy loss caused by EPWS at high flow rates is around 10%.

The change in axial clearance inside the DRT-FM has less effect on the proportion of energy loss caused by EPTD and EPDD and influences the proportion of energy loss caused by EPWS. The increase in axial clearance increases the proportion of energy loss caused by EPWS. The proportion of energy loss caused by EPWS is higher in the 0.65 mm and 0.80 mm groups than in the 0.50 mm group, as shown in Figure 12d.

**Figure 13:**
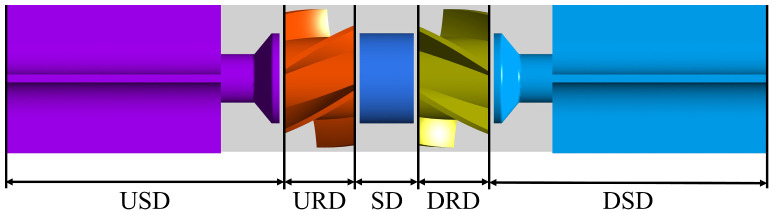
Location of the different domains.

To deeply analyze the effect of clearance variation on the spatial distribution of energy loss in the DRT-FM, the internal flow field of the DRT-FM is divided into five parts, namely, the USD (upstream stationary domain), URD (upstream rotor domain), SD (spacer domain), DRD (downstream rotor domain), and DSD (downstream stationary domain), as shown in Figure 13.

**Figure 14:**
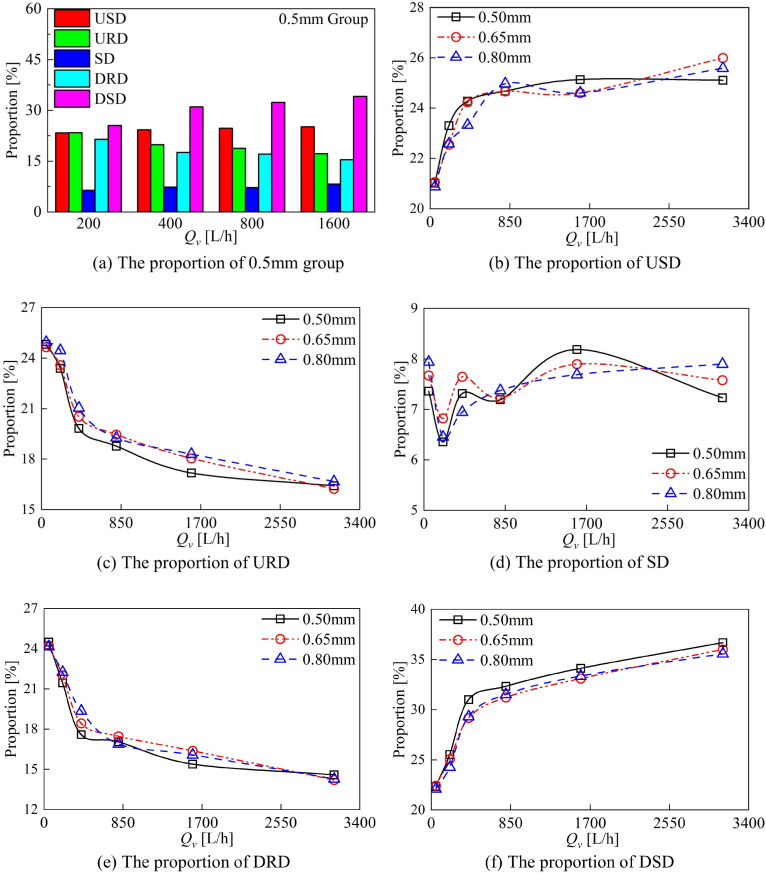
Comparison of the energy loss of different groups in different domains.

The proportion of energy loss in each of the domains within the DRT-FM changes as the flow rate changes, as shown in Figure 14. At low flow rates, the energy loss in the rotor domain is the highest at around 50%, followed by the energy loss in the stationary domain at around 40%, and the spacer domain is the smallest. As the flow rate increases, the proportion of energy loss in the rotor domain decreases to around 35%, the proportion of energy loss in the stationary domain increases to around 60%, and the proportion of energy loss in the spacer domain does not change much with the flow rate. The proportion of energy loss in the DSD is larger than that in the USD, and the proportion of energy loss in the URD is larger than that in the DRD.

When the axial clearance changes, the proportion of energy loss in different domains in each group also changes. With the increase in clearance, the volume of the USD increases, but the energy loss of the USD decreases. The increase in clearance reduces the energy loss in the USD. With the increase in the clearance, the volume of the DSD increases, though the proportion of energy loss in the DSD decreases. The increase in the clearance reduces the energy loss in the DSD. The SD is affected by the upstream and downstream rotors at the same time; the clearance change has a more complex effect on the proportion of energy loss. The proportion of energy loss in the SD is smaller in all domains, and the change in the clearance contributes less to the energy loss of the DRT-FM.

The increase in the axial clearance reduces the proportion of energy loss in the static domain of the DRT-FM and increases the proportion of energy loss in the rotor domain. The effect of axial clearance variation on the proportion of energy loss in the rotor domain is shown in Figure 14c,d. The variation in axial clearance has a similar effect on the proportion of energy loss in the URD and DRD. The proportion of energy loss in the rotor domain is increased in both cases compared to the 0.50 mm group, and the 0.80 mm clearance group has the largest proportion of energy loss in the rotor domain. The increase in axial clearance increases the K factor of the DRT-FM, and the higher the K factor for the same flow rate, the higher the rotor speed, and increasing the axial clearance reduces the energy loss in the rotor domain.

## 5. Conclusions

In this study, we investigated the impact of axial clearance variations on the energy and flow characteristics of a dual-rotor flowmeter (DRT-FM) through numerical simulations. Our findings indicate that increasing axial clearance enhances the meter factor K and reduces Linearity E, thereby improving measurement performance. In the range of 200 L/h to 1600 L/h, the K factors of different groups increase as clearance increases. The K factor of the 0.80 mm group is the largest, showing an average increase of around 6% compared to that of the 0.50 mm group. Additionally, Linearity E decreased, with a minimum of 1.07% in the 0.65 mm group, significantly lower than the 3.33% in the 0.50 mm group. Additionally, increased axial clearance was found to slightly increase pressure loss in the range from 200 L/h to 1600 L/h, with the 0.65 mm group having the largest pressure loss. However, at a flow rate of 1600 L/h, the pressure loss only increases by 0.186 kPa compared to that of the 0.50 mm group. Axial clearance has a greater effect on the pressure loss only at the high flow rate.

Through the analysis of the internal flow field of the double-rotor flowmeter, it is found that the main effect of the axial clearance change is the pressure distribution within the flow field, which reduces the low-pressure region of the upstream and downstream stationary domains, respectively, and the energy dissipation due to the pressure change. Energy loss analysis revealed that at low flow rates, where the flow is laminar, the dominant energy loss is due to the time-averaged velocity gradient, whereas at higher flow rates, turbulent dissipation becomes more significant. Wall shear energy loss is higher at low flow rates but decreases proportionally with increasing flow rates. Increasing axial clearance raises the proportion of energy loss due to wall shear but has a minor impact on energy loss types. It also reduces energy loss in the upstream and downstream static regions, optimizing the DRT-FM’s energy characteristics.

These results suggest that axial clearance variation significantly affects DRT-FM performance, and further experimental and numerical studies are recommended to refine these findings.

## Figures and Tables

**Table 1:** The K factor and linearity E in the range of 200 L/h to 1600 L/h.

Group	*K* Factors (1/L)				Average *K* Factor	Linearity *E*
	200 L/h	400 L/h	800 L/h	1600 L/h		
0.50 mm	2294.48	2181.35	2146.37	2220.69	2210.72	3.33%
0.65 mm	2297.03	2298.82	2313.66	2264.49	2293.50	1.07%
0.80 mm	2373.32	2365.50	2351.06	2285.14	2343.75	1.98%

**Table 2:** The pressure loss of different groups.

Group	Pressure Loss (kPa)				
	200 L/h	400 L/h	800 L/h	1600 L/h	3100 L/h
0.50 mm	0.291	1.018	3.494	12.724	40.336
0.65 mm	0.365	1.196	4.138	12.910	42.918
0.80 mm	0.304	1.050	3.782	11.945	43.798

**Table 3:** The energy loss of different groups.

Group	Energy Loss (W)				
	200 L/h	400 L/h	800 L/h	1600 L/h	3100 L/h
0.50 mm	0.0052	0.0398	0.2823	1.8851	12.4907
0.65 mm	0.0055	0.0406	0.2878	1.9678	12.3527
0.80 mm	0.0054	0.0409	0.2788	1.9262	12.2615

## Data Availability

The data presented in this study are available upon request from the corresponding author.
